# PB.19: Investigation of a novel method for breast discomfort reduction during mammography

**DOI:** 10.1186/bcr3519

**Published:** 2013-11-08

**Authors:** D O'Leary, Z Al Maskari

**Affiliations:** 1 University College Dublin, Ireland

## Introduction

The purpose was to determine the effectiveness of a silicon cushion in providing pain relief during mammography when used to cover sharp edges on the image receptor and/or compression paddle. The impact of the silicon pad(s) on image quality and radiation dose was assessed.

## Methods

The transparent silicon pad was randomly assigned to the right/left breast of the patient; the other breast was imaged as normal. The pad(s) was strategically placed on the mammography machine using three methods. Pain experience data were collected at three discrete time-points during mammography using a visual analogue and Likert scales. Radiologist image evaluators were blinded to pad assignment for image quality evaluation. Radiation dose to the breast was compared with the pad and without.

## Results

Quantitatively, no significant reduction (*P *> 0.05) was observed in the pain experienced due to the silicon pad in either mammographic projection. Qualitatively, there was a trend for pain reduction with the silicon pad. No statistically significant degradation in image quality was assessed in either projection except due to the pad design. There were, however, significant increases in the radiation dose (*P *< 0.00) for both projections due to the slight increase in the compressed breast thickness due to pad thickness.

## Conclusion

The silicon breast cushion requires significant design changes before commercial use for pain reduction intervention in mammography. The study did, however, emphasise that the radiographer plays an important role in the women's experience and communication by the radiographer helps in qualitatively reducing the women's experience of pain during the examination.

